# Trialing addiction neurocircuitry targets and directionality of brain stimulation effects: A deep TMS/fMRI trial in people with alcohol use disorder

**DOI:** 10.1016/j.conctc.2025.101515

**Published:** 2025-06-30

**Authors:** Daniel J. Fehring, Jordan Morrison-Ham, Annalee L. Cobden, Justin Mahlberg, Mengxia Gao, Claire E. Kelly, Arshiya Sangchooli, Devon Stoliker, Emily Giddens, Brody Quinn, Antonia Cholewick, Luiza Bonfim Pacheco, Adeel Razi, Natalia Albein-Urios, Antonio Verdejo-Garcia

**Affiliations:** a School of Psychological Sciences and Turner Institute for Brain and Mental Health, Monash University, Melbourne, Australia; b Monash Biomedical Imaging, Monash University, Clayton, VIC, Australia; c Wellcome Centre for Human Neuroimaging, UCL, London, UK; d CIFAR Azrieli Global Scholars Program, CIFAR, Toronto, ON, Canada; e Discipline of Psychology, Federation University, Berwick, VIC 3806, Australia

**Keywords:** Deep transcranial magnetic stimulation (dTMS), Alcohol use disorder (AUD), Transcranial magnetic stimulation (TMS), Functional magnetic Resonance imaging (fMRI), Spectral dynamic causal modeling (spDCM)

## Abstract

**Background:**

Excessive alcohol consumption is a global health concern, with an estimated 400 million people living with alcohol use disorder (AUD). Current treatments for AUD have limited efficacy and fail to address its diverse neurobiological underpinnings. There are at least two cortico-striatal circuits relevant to AUD neurobiology: a weakened dorsolateral prefrontal cortex (dlPFC) pathway, and a heightened ventromedial prefrontal cortex (vmPFC) pathway.

**Purpose:**

This trial aims to examine whether deep transcranial magnetic stimulation (dTMS) can recalibrate the neurocircuitry disrupted in AUD as a proof-of-concept for its therapeutic potential. We will assess the capacity of two theta-burst stimulation protocols to modify neuroimaging and behavioral indices of AUD-related neurocircuitry alterations.

**Methods:**

We will conduct a randomized, single-blind, sham-controlled crossover trial with 30 adults with moderate to severe AUD (aged 18–49). Participants will receive two doses of active or sham dTMS (for 2 sessions; 7 days apart; order counterbalanced) targeting the dlPFC or vmPFC with intermittent or continuous theta-burst stimulation, respectively.

**Results:**

Primary, secondary, and exploratory outcomes (i.e., stimulation-induced changes in neural circuit connectivity, executive control/decision-making, and craving-related emotions, respectively) will be collected before and after each dTMS dose. Additional exploratory outcomes (daily craving experiences and weekly alcohol consumption) will be collected across a 90-day period from the first session.

**Discussion:**

This trial innovates by utilizing distinct dTMS approaches to specifically target two functionally segregated neurocircuitries disrupted in AUD. Results will inform the development of a larger-scale trial by establishing optimal therapeutic approaches for AUD.

## 1 Introduction

### 1.1 Background and rationale

Globally, excessive alcohol consumption contributed to 2.6 million deaths in 2019, with an estimated 400 million people (7 % of the world's population aged 15 years or older) living with alcohol use disorder (AUD) [1]. In Australia, 1 in 3 people (31 %) aged 14 or over have reported drinking alcohol at levels that put them at increased risk of harm [2]. Treatments for AUD are only accessed by a small percentage of those living with the disorder, with only 7.7 % of those meeting the criteria for a 12-month diagnosis seeking treatment [3]. Barriers to treatment include stigma, a lack of motivation to change, and the limited efficacy of approved pharmacological treatments [4,5]. Moreover, although these pharmacological approaches address certain neurobiological aspects of AUD, particularly at the level of neurotransmitter systems [6], they do not necessarily target the altered brain networks that drive and sustain the disorder. This network-level dysfunction, encompassing disrupted neural circuits that underlie key processes such as reward, craving and executive control, remains inadequately addressed. Thus, current treatments may be less effective at addressing the diverse biotypes of AUD [7], and contribute to a higher risk of relapse. There is therefore an urgent need for interventions that specifically target these altered networks to improve patient outcomes and quality of life for those with AUD.

Transcranial magnetic stimulation (TMS), a form of non-invasive brain stimulation, has been proposed as a therapeutic intervention in AUD due to its ability to directly modulate the aberrant neural circuitry underlying the disorder [8]. TMS depolarizes neurons and alters neuronal excitability by inducing electromagnetic currents in the superficial layers of the brain [9], to potentially modulate behaviour [10]. Theta-burst stimulation, a form of repetitive TMS, more closely mimics endogenous neural firing patterns, and demonstrates increased efficacy in augmenting neuronal activity and behavior than fixed-frequency repetitive TMS [11]. Theta-burst stimulation can be administered using distinct protocols with varied parameters to selectively modulate neuronal function: intermittent theta-burst stimulation (iTBS) typically produces an increase in neuronal activity, while continuous theta-burst stimulation (cTBS) reduces activity [12]. Importantly, these TMS protocols demonstrate clinical efficacy in various neuropsychiatric and neurological disorders, and are currently approved for treating various forms of major depressive disorder [13], obsessive compulsive disorder [14], and for smoking cessation [15].

Previous studies examining therapeutic TMS in AUD demonstrates its potential for reducing craving and alcohol use, with small to moderate effect sizes [[16], [17], [18]]. Yet most studies have focused solely on stimulation of the dorsolateral prefrontal cortex (dlPFC), due to its involvement in governing executive functions that are critically disrupted in AUD [19,20]. However, given the complex and varied nature of AUD, it is likely that there are multiple nodes that could be stimulated for therapeutic benefit. Central to AUD neurobiology are at least two disrupted and dissociable cortico-striatal circuits associated with executive and limbic control, mediated by the dlPFC and vmPFC, respectively. The dlPFC, implicated in executive control, exerts top-down regulation through projections to dorsal striatal regions. Dysfunction in this lateral/dorsal pathway has been linked to impaired inhibitory control processes, which are critical for regulating alcohol use [21]. Impaired dlPFC functioning also correlates with disruptions in goal-directed behaviors, and altered functional connectivity between the dlPFC and dorsal striatal nodes predicts levels of alcohol consumption and cognitive deficits [22]. Conversely, the vmPFC is central to the medial/ventral circuit, which maintains strong reciprocal connections with the ventral striatum and insula [23,24]. The vmPFC plays a pivotal role in incentive valuation and value-based decision-making, processes that are markedly impaired in AUD [25]. Dysfunction within this circuit is associated with heightened impulsivity, exaggerated responses to alcohol-related cues, and impaired stress regulation [25]. Notably, hypoactivity in the vmPFC and insula during stress predicts increased alcohol use severity following relapse [21]. Importantly, these circuits can be differentially modulated by targeted neuromodulation [26], and thus represent promising yet dissociable therapeutic targets. By diversifying target regions and stimulation protocols, we may be able to better address the heterogeneity of AUD, optimizing treatment responses and reducing relapse risk. By applying this expanded framework, we may create more comprehensive and adaptive interventions that better align with the complex pathophysiology of AUD.

Although prefrontal TMS has demonstrated promising results, its effectiveness may be limited by the inability to reach these critical subcortical connections with the use of traditional figure-eight coils. The development of wide-volume TMS approaches, including ‘deep TMS’ (dTMS), addresses this limitation by enabling direct modulation of a wider range of cortical and subcortical nodes through stimulation of larger volumes of neuronal tissue [27]. Wide-volume TMS generates a stronger and less focal electromagnetic field (e-field); in particular, Brainsway dTMS has been shown to stimulate brain regions up to 5 cm beneath the skull [28,29], through the use of coil windings within a helmet-like device (H-coil) worn by the patient. In comparison, traditional figure-eight coils only stimulate superficial layers of the brain, approximately 1.5 cm from the middle of the coil [28]. Modeling of dTMS e-fields demonstrates the superiority of the H-coil when targeting deeper regions of the prefrontal cortex. Computational modelling demonstrated that a figure-eight coil over the prefrontal cortex could stimulate the medial prefrontal cortex at only 35 % of the maximal e-field intensity, while the H-coil can stimulate both the dorsolateral and medial prefrontal cortex at up to 70 % of the maximal intensity, enabling deeper neural modulation while maintaining safe stimulation levels [30]. This enhanced capacity of dTMS to target both cortical and subcortical structures allows us to specifically target these two functionally segregated neurocircuitries disrupted in AUD, providing a more comprehensive and effective approach than figure-eight TMS. Indeed, emerging evidence from pilot trials in individuals with AUD suggests that high-frequency dTMS using the H7 coil can modulate resting-state functional connectivity deficits and reduce alcohol consumption [31], supporting its potential as a neuromodulatory intervention in this population. Moreover, delivering dTMS using an accelerated protocol, defined as delivering multiple sessions per day over a short period, allows us to shorten intervention duration and improve response time whilst maintaining comparable efficacy and safety [32]. To further characterize the influence of dTMS on these disrupted neurocircuits, we will apply spectral dynamic causal modeling (spDCM) on resting-state fMRI data to measure effective connectivity, which indexes the valence (excitatory or inhibitory) and directionality (input and output) of neural connections between nodes of these targeted neurocircuits [33], enabling detailed insights into how dTMS influences the directionality and strength of neural information flow in AUD.

### 1.2 Objectives

We aim to examine whether dTMS can recalibrate the neurocircuitry disrupted in AUD as proof-of-concept of its therapeutic potential. Specifically, we will assess the capacity of two promising TBS protocols targeting the dlPFC and vmPFC to modify neuroimaging and behavioral indices of AUD-related neurocircuitry alterations. The primary outcome will be stimulation-induced changes in neuroimaging indices of effective connectivity within the targeted neural circuits. The secondary outcome will be stimulation-induced changes in a cognitive test battery measuring executive control and value-based decision-making. Exploratory outcomes will include stimulation-induced changes in interoceptive processes associated with craving indicated by laboratory tasks, and fluctuations in daily craving and alcohol consumption over time (90 days) measured via experience sampling.

## 2 Methods

### 2.1 Trial design

We will use a randomized, single-blind, sham-controlled crossover design to compare two distinct dTMS protocols. Neuroimaging, cognitive, and interoception indices will be collected before and after each dTMS intervention, to assess AUD-related neurocircuitry alterations. Data related to craving and alcohol use will be collected longitudinally across a 90-day period.

### 2.2 Participants

Thirty adults, aged 18–49 years, who endorse current moderate to severe alcohol use disorder according to DSM-5 criteria. Table 1 describes full eligibility criteria. Participants will provide informed written consent for each aspect of the trial and be assigned a pseudo-randomized ID to ensure confidentiality throughout the trial.

**Table 1:** Inclusion and exclusion criteria.

Inclusion	Exclusion
● Meet criteria for moderate-severe alcohol use disorder (QuickSCID-5) ● Ages 18-49 ● Willing to abstain from alcohol 36 h prior to sessions, and any other illicit substance for 7 days^a^	● Meet criteria for current and/or past psychosis, mania, or major unstable psychiatric disorder, such as bipolar and schizophrenia (QuickSCID-5) ● Self-reported history of seizure/epilepsy or first-degree family history of epilepsy ● Brain trauma with loss of consciousness in the past 6 months, ● History of regular severe headaches and/or migraines ● Hearing impairments likely to be exacerbated by brain stimulation ● Severe cognitive impairment (Montreal Cognitive Assessment [MoCA]) ● Currently taking high doses of benzodiazepines (>20 mg diazepam or equivalent) ● Currently pregnant/breastfeeding ● MRI contraindications such as ferromagnetic metal in the head, brain surgery, pacemaker ● Previous adverse reactions to brain stimulation ● Serious/unstable medical condition or illness

a Where participants are asked to attend a session less than 36 h prior to testing or accidentally drink, participants will still be included if they have had 0 standard drinks 12 h prior to the session, ≤3 standard drinks in the past 24 h, and blood alcohol concentration of 0.00 on the day of testing.

### 2.3 Recruitment, screening and randomization

#### 2.3.1 Recruitment and screening

Participants will be recruited through advertisements across various online platforms using targeted ads. Prospective participants will initially be screened online using the AUDIT [34] (cutoff score of 15), with moderate-severe AUD diagnosis (as well as mental health and neurological exclusions; see Table 1) subsequently confirmed using the QuickSCID-5 via a Telehealth interview. In addition, participants will be screened for contraindications for MRI and dTMS (see Table 1), and complete the Montreal Cognitive Assessment (MoCA) [35] to determine the presence of severe cognitive impairment to confirm the capacity for informed consent (cutoff score of 10).

#### 2.3.2 Blinding and randomization procedures

Randomization into one of two trial arms (iTBS or cTBS; see *2.4*) with an allocation ratio of 1:1 will occur via a generated allocation sequence stratified on sex at birth (female, male, intersex) and AUD severity (moderate, severe; indexed by the QuickSCID-5) to ensure an equal distribution in each stimulation arm. Participants will be randomly counterbalanced to receive either active or sham theta-burst stimulation in the first session, and the opposite stimulation condition in the second session. Only the dTMS clinicians will have access to the randomization to administer the appropriate dTMS protocol. dTMS clinicians will not administer any outcome measures. Participants and outcome assessors will be blinded to the participant's intervention condition. The sham stimulation condition will match the parameters of the active condition to preserve blinding to the participants and outcome assessors. In the case of an adverse event, blinding procedures will no longer be followed, and first aid and risk management strategies will be applied.

### 2.4 Interventions

Following randomization into one of two trial arms (iTBS or cTBS), participants will attend two accelerated dTMS sessions separated by one week (±3 days). In one session, both dTMS doses will be active, while in the other session both doses will be sham (counterbalanced). In each accelerated dTMS session, participants will receive two doses of theta-burst dTMS (iTBS via H1 coil or cTBS via H7 coil; delivered by Brainsway 104 system, Brainsway, Jerusalem, Israel) with 50 min between each dose, in accordance with the standard FDA-approved clinical application of accelerated TMS protocols [36]. Although deep TMS coils stimulate relatively large volumes of neuronal tissue, the H1 and H7 coils were selected for their field distributions that are centred over the key network nodes of interest [31,37]. Specifically, the H1 coil produces a broad field primarily over the lateral prefrontal cortex, enabling modulation of the dlPFC, while the H7 coil predominantly stimulates the medial prefrontal and anterior cingulate cortex, allowing modulation of the vmPFC [37]. Given that the H1 coil does not have a sham mode and participants are naïve to stimulation, sham stimulation for both the iTBS and cTBS conditions will be administered using the H4 coil in sham mode. Before the first dose of dTMS in a given session (active or sham; order counterbalanced), resting motor threshold (RMT) assessment will be conducted following standardized procedures [38]: RMT will be calculated using muscles in the hand (H1 coil for iTBS, and H4 coil sham) or the foot (H7 coil for cTBS). Additionally, before the first dose of dTMS in each session, a short stimulation adaptation procedure will be applied, to ensure participants are comfortable with the given stimulation. The adaptation procedure will involve exposing the participant to a short train (∼2 s for iTBS, 4 s for cTBS) of the dTMS protocol at a reduced intensity to acclimate the participant. Participants are encouraged to concentrate on slow, intentional breathing to mitigate anxiety during the stimulation [39].

#### 2.4.1 iTBS intervention

To recalibrate the lateral/dorsal circuit disrupted in AUD, iTBS over the dlPFC will be applied to increase top-down DLPFC-striatum inhibition. iTBS will be administered using the H1 Brainsway coil targeting the dlPFC, positioned 6 cm anterior to the motor hotspot, using standardized localization procedures [38,40]. Stimulation will be administered at 100 % of the hand RMT.

The iTBS protocol will consist of three-pulse bursts at 50Hz repeated every 200 ms (5Hz) for 2 s, with an 8-s intertrain interval, for 40 trains. The total stimulation time will be 380 s (1200 pulses) [41].

#### 2.4.2 cTBS intervention

To recalibrate the medial/ventral circuit disrupted in AUD, cTBS over the VMPFC will be applied to reduce excitation in the VMPFC-striatum pathway. cTBS will be administered using the H7 Brainsway coil targeting the vmPFC positioned 4 cm anterior to the motor hotspot using standardized localization procedures [42]. Stimulation will be administered at 80 % of the foot RMT.

The cTBS protocol will consist of two trains of a standard cTBS sequence (as per Huang [12]), with a 30-s intertrain interval. Each train will consist of three-pulse bursts at 50Hz repeated every 200 ms (5Hz) for 40 s (600 pulses). The total stimulation time will be 110 s (1200 pulses).

#### 2.4.3 Sham intervention

Across all participants, sham stimulation will be administered using the H4 coil in sham mode, which replicates the acoustic and scalp sensations of active stimulation without generating effective field penetration into the brain [43]. The H4 helmet will be positioned in the same location as during active stimulation (e.g., 6 cm anterior to the motor hotspot for iTBS sham) and delivered at the same intensity relative to the coil motor threshold.

### 2.5 Outcomes

**Fig. 1:**
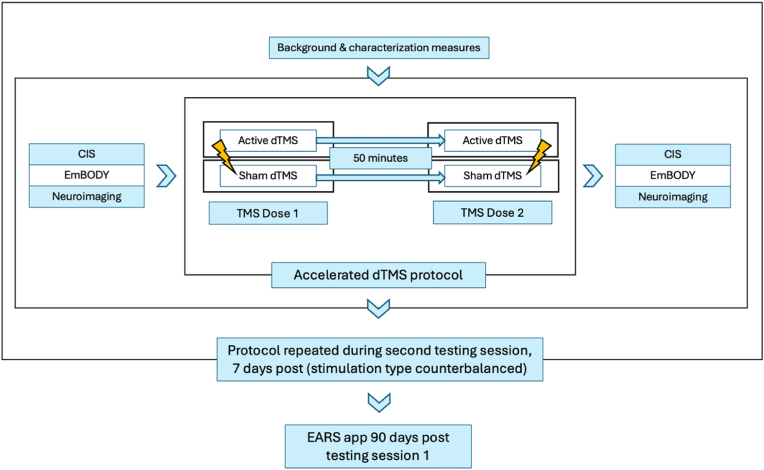
Flowchart of testing day procedures. Participants will be asked to attend two testing sessions, 7 days apart (±3 days). The type of dTMS they receive on a given day will be randomized and counterbalanced. The protocol will be the same across both testing sessions. The EARS application will be installed in the first session, and collect craving and alcohol use longitudinally (90 days).

Neuroimaging indices of effective connectivity (primary outcome), executive control and decision-making (secondary outcome), and interoception (exploratory outcome) will be assessed directly prior to the first dTMS dose and immediately after the second dTMS dose, in each session. Craving and alcohol use (exploratory outcomes) will be collected longitudinally (90 days following the first session). Background and characterization measures will be collected at the beginning of each session prior to dTMS delivery (Fig. 1).

#### 2.5.1 Primary outcomes

##### 2.5.1.1 Neuroimaging

All MRI data will be collected on a Siemens 3T Skyra MR machine with a 64-channel head coil, with physiological recording included for the resting-state fMRI scans. Functional imaging for spDCM analyses [44] will be performed using multi-band multi-echo imaging to enhance the signal-to-noise ratio of deep brain structures during resting-state scans. The imaging parameters are as follows: multiband factor = 4, number of slices = 40, repetition time (TR) = 910 ms, echo times (TEs) = 13.0, 30.0, 46.0, 63.0 ms, flip angle = 56°, slice thickness = 3.2 mm, interpolated voxel size = 3.2 × 3.2 × 3.2 mm^3^, field of view (FoV) = 206 × 206 mm, and acquisition time = 8 min. During resting-state scans, participants will be instructed to remain relaxed with eyes open directed towards a fixation stimulus. Task-fMRI data will also be collected while participants complete a craving task using the Alcohol Cue Reactivity Paradigm [45], during which they will view images of alcohol-related stimuli and rate their level of craving, presented according to typical blocked cue-reactivity paradigms [46].

Within the same scanning session, additional sequences will be acquired to assist functional imaging analysis or for secondary analyses, including structural T1-weighted scans (TR = 2300 ms, TE = 2.07 ms, flip angle = 9°, slice thickness = 1.0 mm, interpolated voxel size = 1.0 × 1.0 × 1.0 mm^3^, FoV = 256 × 240 mm, matrix 256 × 256, acquisition time = 5 min), T2-weighted scans (TR = 3200 ms, TE = 452 ms, slice thickness = 1.0 mm, voxel size = 1 × 1 × 1 mm^3^, FOV = 256 × 256 mm, matrix = 256 × 256, acquisition time = 4 min) and diffusion-weighted scans (71 diffusion gradient directions with a b-value = 3000 s/mm^2^, 1 b = 0 s/mm^2^ image, 56 axial slices, TR = 5900 ms, TE = 171 ms, slice thickness = 2.5 mm, 2.5 mm isotropic voxels, matrix size = 86 × 86, FoV = 212 × 212 mm, acquisition time = 8 min).

For both the functional and diffusion MRI sequences, additional images will be acquired with matched parameters except for opposite phase encoding direction (L > R versus R < L) to correct for susceptibility distortions.

#### 2.5.2 Secondary outcomes

##### 2.5.2.1 Executive control/decision-making: Cognitive Impulsivity Suite

We will assess behavioural changes in executive/inhibitory control and value-based decision-making using a shortened version of the Cognitive Impulsivity Suite (CIS) [47]. The CIS is a cognitive test battery comprising three tasks. These tasks assess different cognitive pathways leading to impulsive actions and choices, namely, attentional control, information gathering (prior to decision-making), and reward monitoring/shifting facets. It has shown excellent test-retest reliability and sensitivity to change, as well as ability to detect individual differences linked to AUD [45]. A shortened 2 blocks per task, instead of the standard 4 blocks, will be administered to aid the proximity of each task to stimulation time while maintaining the validity of the tasks (i.e., trials per block consistent with the full test battery). The attentional control task (bounty hunter task) will consist of 60 trials per block, and the information gathering (caravan spotter) and monitoring/shifting (prospectors gamble) tasks will each have 40 trials per block.

#### 2.5.3 Exploratory outcomes

##### 2.5.3.1 Interoceptive sensing of craving-related emotions: EmBODY

The EmBODY tool uses visual bodily sensation maps and assess interoceptive sensing by presenting different emotions and allowing participants to denote where on the body they feel the emotion most or least intensely [48,49]. A shortened version of the EmBODY [50] tool will be administered to explore whether dTMS influences the interoceptive sensing of craving and related emotions including desire, lack of control, and relief.

##### 2.5.3.2 Daily craving and weekly alcohol consumption over time: experience sampling

We will ask participants to install a validated ecological momentary assessment application called Effortless Assessment Research System (EARS; KSANA) on their phone [51]. Visual analog scales related to craving intensity will be administered three times a day, pseudo-randomized within morning (8–10 a.m.), afternoon (3–5 pm), and evening (7–9 pm) blocks for 90 days following the first session. Participants will have a 2-h window to respond to survey notifications. Alcohol consumption will be assessed once per week.

#### 2.5.4 Background and characterization measures

We will obtain phenotypic data from participants to gain a better understanding of the sample. The Timeline Follow-Back will be used to measure alcohol consumption in the last month [52]. Hair samples will be collected to gain objective measures of alcohol use over the same period. Participants will also complete the Obsessive Compulsive Drinking Scale (OCDS) [53], Penn Alcohol Craving Scale (PACS) [54], and Fagerström Test for Nicotine Dependence to characterize alcohol and tobacco use patterns [55]. Heartbeat detection [56], Two-Stage [57] and Horizons [58] tasks will be used to assess AUD-related cognitive/affective mechanisms. The World Health Organization Quality of Life Brief Version (WHOQOL-BREF) [59] will be used to assess quality of life, and the Depression Anxiety Stress Scale 21 (DASS-21) to assess depression and anxiety [60].

### 2.6 Session Overview

Upon arrival for the session, participants will be provided with a full trial description (including descriptions of the MRI and TMS procedures) and complete informed written consent. In addition, participants will be breathalyzed to confirm a blood alcohol concentration (BAC) of 0.00. Participants will again be screened for dTMS and MRI contraindications to ensure they do not meet any transient exclusion criteria (e.g., headache or migraine) for the given session, in addition to completing the Clinical Institute Withdrawal Assessment Alcohol Scale Revised (CIWA-AR) before each dTMS dose. A withdrawal severity score below 10 has consistently been considered safe for administering TMS in individuals with AUD, based on established protocols and safety guidelines from prior studies [[61], [62], [63]]. Neuroimaging (primary outcome), executive control/decision-making (secondary outcome; indexed by the CIS), and interoceptive processing (exploratory outcome; indexed by EmBODY) outcomes will be assessed directly prior to the first dTMS dose, and immediately after the second dTMS dose, in each session (Fig. 1). Participants will attend two accelerated dTMS sessions separated by one week (±3 days). Fig. 1 illustrates the overall flow of the testing session procedures.

### 2.7 Data analysis

Preliminary analyses will determine whether, despite randomization, the intervention arms are unbalanced on each potential confounder. Normality will be assessed using skewness and kurtosis, with values between ±1.5 and ± 2, respectively, considered normal. For non-normal variables, suitable transformations (e.g., log transformation) will be applied, and geometric means with 95 % confidence intervals will be reported. Primary outcome measures will be determined by a stimulation-induced change in the neuroimaging measures (see 2.7.1), and secondary outcome measures by the CIS. The within-subject effect of the dTMS intervention to influence different aspects of AUD symptomatology (CIS, and exploratory outcomes) will be evaluated using linear mixed models with session-intervention interactions, using restricted maximum likelihood estimation and optimal covariance structure based on AIC/BIC. This approach allows for 1) the direct testing of dTMS treatment effects, as well as potential order effects, 2) account for the fact that repeated observation from the same subjects are correlated, 3) and may include relevant covariates (e.g., demographics or those from the exploratory measures) [64]. Between-group (iTBS vs. cTBS) comparisons will be analyzed using mixed-model regression analyses, adjusting for baseline measures and confounders. Moreover, relationships between primary, secondary, and exploratory outcomes and background and characterization measures will be explored through mixed-model regression analyses. An intention-to-treat (ITT) approach will be used, with missing data imputed via multiple imputation. All frequentist tests will be two-tailed with alpha set at 0.05.

#### 2.7.1 Neuroimaging analyses

Primary neuroimaging outcomes will be changes in effective connectivity, indexed by spDCM of fMRI data, within the two cortico-striatal circuits disrupted in AUD and targeted here using dTMS: (1) the lateral/dorsal circuit, involving the dlPFC and dorsal striatal regions including the dorsal caudate, and (2) the medial/ventral circuit, involving the vmPFC and the insula. These changes will be assessed in relation to: 1) Stimulation Protocols (Between-Subjects): Differences in effective connectivity resulting from iTBS vs. cTBS, and 2) Stimulation Effects (Within-Subjects): Changes in effective connectivity pre- and post-dTMS. Firstly, all functional images will be preprocessed using state-of-the-art pipelines including slice-timing correction, realignment, registration to Montreal Neurological Institute standard space [65] and spatial smoothing. Participants with excessive head motion will be excluded from subsequent analyses. Then, at the individual level, we will apply spDCM to estimate effective connectivity parameters for each participant under each condition (active vs. sham). At the group level, a two-stage hierarchical parametric empirical Bayes (PEB) [66] analysis will be conducted: 1) First Level (Within-Subjects): Assess within-subject connectivity changes by comparing pre- and post-dTMS scans during active versus sham sessions. This will evaluate the immediate effects of dTMS and differentiate between active and sham stimulation effects within participants, and 2) Second Level (Between-Subjects): Compare between-group differences (iTBS vs. cTBS) in the within-subject connectivity changes observed during active and sham sessions, to determine the differential impacts of the stimulation protocols. Effective connectivity changes between conditions that exceed the significance threshold of posterior probability >0.95 will only be reported (equivalent to a strong evidence).

### 2.8 Oversight, monitoring, and reimbursement

#### 2.8.1 Adverse events

If an Adverse Event (AE) is observed during the face-to-face sessions, or reported after the session, after establishing appropriate health and safety measures and implementing risk controls, the Principal Investigator (PI) will be immediately notified. The PI reviews each AE reported in consultation with the PI team, including first-aid trained personnel and licensed healthcare professionals. All AEs will be logged and documented within REDCap, and the trial program team is informed so they can adjust any measures appropriately in response to the AE. All AEs deemed 1) unexpected; 2) possibly or probably related to trial participation; and 3) suggest increased risk for trial participants, will be reported to the Monash University Human Research Ethics Committee within seven days of the AE.

#### 2.8.2 Protocol fidelity and participant adherence

To ensure the dTMS intervention is delivered consistently and as intended, a series of protocols have been established. First, stimulation protocol fidelity will be maintained by fixed stimulation parameters set on the Brainsway device. Localization fidelity will be maximized and monitored by (i) dTMS administration by expert operators, (ii) using standardized localization protocols [38,40,43], and (iii) random session audits by the chief dTMS clinician. Participant adherence to and tolerability of the dTMS will be monitored via attendance and standardized post non-invasive brain stimulation interventions. The fidelity of all other outcomes will be maintained through the automated administration of tasks in REDCap, which branches according to randomization, with data quality and completion audited by the clinical trial lead.

#### 2.8.3 Participant reimbursement

Participants will receive compensation in the form of gift cards at various points during the trial. They will be provided: (1) $150 for each in-person experimental session, (2) $30 upon completing a cognitive task battery, and (3) daily incentives for application (EARS) engagement, calculated at $2 per day with a potential maximum of $180 over the 90 day period. Participants will also receive an up to $100 bonus scaled on their overall percentage of engagement with the EARS application. These reimbursements are designed to reflect the time and effort required for trial participation. Additionally, transportation services (e.g., taxi) will be offered to ease participant burden of attending in-person sessions.

### 2.9 Data sharing and dissemination

Trial results will be shared primarily through publications and with key stakeholders and collaborators. Since this trial serves as a preliminary phase for a larger clinical trial, data cannot be shared at this stage. However, data will be made openly available after completion of its parent project.

## 3 Discussion

Targeting the different neural mechanisms underlying AUD may improve treatment outcomes for this chronic and relapsing condition. There is emerging evidence that TMS can be used to change neural connectivity in brain regions related to AUD pathology [67,68], as well as craving and alcohol use outcomes [16,61,69,70]. However, previous studies have primarily focused on the dlPFC as the target stimulation region, using standard figure-eight coils [16,61,68,70]. In contrast, the current protocol was designed to overcome these limitations by leveraging dTMS to achieve deeper and more extensive activation of the targeted brain regions, and to asess flow-on effects on directed neural network connectivity. Specifically, here we target two functionally segregated neurocircuitries disrupted in AUD (dlPFC-dorsal striatum and vmPFC-insula-ventral striatum), addressing the potential involvement of multiple dysregulated nodes within the network of AUD that could be stimulated for therapeutic benefit.

This trial will build upon the current literature examining TMS for AUD, and will be the first to utilize a novel, parallel groups design to examine the effects of theta-burst dTMS to two different brain networks disrupted in AUD. In addition, the use of a crossover design provides the opportunity to examine the direct effects of the dTMS (active vs sham) within each stimulation arm (iTBS vs cTBS) across a range of primary, secondary, and exploratory outcomes (stimulation-induced changes in neural effective connectivity, cognition, and emotion/interoception processes, respectively). Finally, exploratory measures to identify how dTMS affects craving and alcohol consumption over time will provide further insights into the interaction of dTMS and AUD symptomatology. Results from this trial are expected to contribute to the development of larger-scale, randomized controlled trials, and establish optimal therapeutic profile patterns for those with AUD.

## CRediT authorship contribution statement

**Daniel J. Fehring:** Writing – review & editing, Writing – original draft, Supervision, Project administration. **Jordan Morrison-Ham:** Writing – review & editing, Writing – original draft, Visualization. **Annalee L. Cobden:** Writing – review & editing, Writing – original draft. **Justin Mahlberg:** Writing – review & editing, Supervision, Project administration, Funding acquisition. **Mengxia Gao:** Writing – review & editing, Supervision, Project administration. **Claire E. Kelly:** Writing – review & editing. **Arshiya Sangchooli:** Writing – review & editing. **Devon Stoliker:** Writing – review & editing. **Emily Giddens:** Writing – review & editing. **Brody Quinn:** Writing – review & editing. **Antonia Cholewick:** Writing – review & editing. **Luiza Bonfim Pacheco:** Writing – review & editing. **Adeel Razi:** Writing – review & editing, Project administration, Methodology, Funding acquisition, Conceptualization. **Natalia Albein-Urios:** Writing – review & editing, Supervision, Methodology, Funding acquisition, Conceptualization. **Antonio Verdejo-Garcia:** Writing – review & editing, Writing – original draft, Supervision, Project administration, Methodology, Funding acquisition, Conceptualization.

## Ethics

The trial protocol has been approved by the Monash University Human Research Ethics Committee (MUHREC ID: 42604), and will be conducted in line with the Declaration of Helsinki statement of ethical principles for medical research.

## Data availability

No data was used for the research described in the article.

## Funding information

This study is funded by the Untangling Addiction Program of the Wellcome Leap. AVG is funded by an NHMRC Investigator Grant (2009464).

## Declaration of competing interest

The authors declare that they have no known competing financial interests or personal relationships that could have appeared to influence the work reported in this paper.
